# Effect of Propolis Preparations on Transepithelial Electrical Potential, Resistance, and Ion Transport in* In Vitro* Study

**DOI:** 10.1155/2019/3756092

**Published:** 2019-01-15

**Authors:** Paulina Smyk, Iga Hołyńska-Iwan, Dorota Olszewska-Słonina

**Affiliations:** ^1^Department of Pediatric Nursing, Faculty of Health Sciences, Collegium Medicum in Bydgoszcz Nicolaus Copernicus University in Torun, Poland; ^2^Department of Pathobiochemistry and Clinical Chemistry, Faculty of Pharmacy, Collegium Medicum in Bydgoszcz Nicolaus Copernicus University in Torun, Poland

## Abstract

**Background:**

Propolis and its ethanol extract show positive germicidal, bacteriostatic, and anti-inflammatory antioxidants and regenerative properties after use on the surface of the skin. Propolis is in common use in production of cosmetics and in folk medicine. The influence of this resinous mixture on ion channels, channels located in skin cells membranes and skin electrical resistance, was not explained.

**Objective:**

The main aim of the study was the evaluation of electrophysiological skin parameters during mechanical and chemical-mechanical stimulation after use of ethanol extract of propolis and propolis ointment in comparison with iso-osmotic Ringer solution.

**Methods:**

Skin fragments were taken from white New Zealand rabbits and distributed into three experimental groups which were incubated in ethanol extract of propolis (EEP), propolis ointment, and Ringer solution. Then they were placed in a Ussing chamber to measure electrophysiological parameters values.

**Results:**

In this study the influence of EEP on changes in value of electrical potential during block of chloride ions transport at the same time was observed. Ethanol propolis extract dissolved in water increases the transepidermal sodium ions transport in contrast to propolis ointment.

**Conclusion:**

The way of preparation cosmetics, which contain propolis, has effects on transepidermal ions transport in the rabbit's skin. The value of skin electrical resistance is changing with penetration depth of active propolis substances contained in cosmetics.

## 1. Introduction

Propolis has been used in medicine and cosmetology for centuries. The name of propolis comes from the Greek language, from the words pro- and polis-, which means the city's rampart. The propolis is a complex material collected by honeybees from buds, leaves, and parts of trees or other plants. This substance is a viscous, sticky, resin-balsamic mass. Bees used the propolis to strengthen the construction of the hive, by sealing its interior for the protection against microorganisms [1–3].

In Poland the propolis is obtained from leafy trees (poplar, birch, alder, oak, willow, and chestnut) and coniferous trees (fir, pine, and spruce). The chemical composition of propolis depends upon geographical origin, plants, and change of climate and upon the species of bees. It has a few colours which depend on its source and age [4]. Propolis activities depend on various compounds which it contains. The most biologically active substances are scarcely soluble in oil, water, or other solvents. Propolis should not be used as a crude material [5]. Ethanol extraction is the most popular technique for the production of propolis extracts. EEP has limited uses in cosmetology [6]. In ethanol extract of propolis there is the greatest in quantity of flavonoids. Besides, aromatic acids and esters of aromatic acids were also found. The content of flavonoid compounds in EEP is 2,72-10,81% according to Kędzia et al. and Ellnain-Wojtaszek et al. The average content of flavonoid compounds in EEP in Polish propolis was 5% [7, 8].

Propolis and its extracts have long been used for the prevention and treatment of a variety of diseases due to its antibacterial [9–13], antiviral [14], antifungal, antioxidant [12, 15–18], anesthetizing, cytostatic [19, 20], anti-inflammatory [12, 20], immune-strengthening [19], and hepatoprotective properties [19, 21]. Ethanol extract of propolis is widely used in cosmetics. Although propolis is also in use in the treatment of skin diseases caused by microorganisms, such as folliculitis, sweat gland infections, boils, impetigo, nodules, and pyodermas, as well as the treatment of fungal and viral diseases [22].

Propolis is quite safe specimen, but it also causes allergic reactions. Persons with a tendency to be allergic to other bee products (honey/bee pollen) should be especially attentive. After topical application of propolis, indicated by the following symptoms: redness, swelling, and itching of the skin [23], hypersensitivity reactions may occur. In medical literature many publications have centred on the transepithelial ion transport in various tissues (trachea, caecum) [24–28], skin of amphibians [29], and propolis properties, but there are no publications about effect of propolis on ion channels located in the skin cell membranes. Modified Ussing's method used in this experience facilitated the analysis of the interaction of propolis preparations with transepithelial ion transport, value of electrical potential of epithelial tissue and its changes before and during stimulation, and integrity and viability of epithelial tissue.

## 2. Materials and Methods

The 62 skin fragments taken from 4 experimental animals of both sex were located in the modified Ussing chamber.

Experimental material was divided into three groups, only special rules:

I group: Long-term incubation in 1 ml ethanol extract of propolis dissolved in 100 ml water (N =22)

II group: Undisturbed ion transport (N = 22)

III group: Long-term incubation in propolis ointment (N = 18)

The amount of experimental material (fragments of the rabbit's skin) from females was equal to the material from males in each group.

The Ussing method used in this experiment allows evaluating

- transepithelial electrical potential differences in stationary conditions before and after stimulation (PD, mV);
- changes of electrical potential differences during stimulation (dPD, mV);
- minimal transepithelial electrical potential (PDmin, mV);
- maximal transepithelial electrical potential (PDmax, mV)
- transepithelial resistance (R, Ohm/cm^2^).

The procedure protocol for three experimental groups during preincubation (30 minutes) and short-term mechanical and chemical stimulation was presented in Table 1.

### 2.1. Animals

Skin samples were taken from experimental animals (adult, New Zealand rabbits of both sexes, two males and two females, from animal husbandry in Medical University of Silesia in Katowice, weighting between 3.5 and 4.0 kilograms, three to four months old). The animals were maintained on a standard light/dark cycle: 12 hours light and 12 hours night schedule. The rabbits were anesthetized with high concentration of isoflurane in carbon (IV) dioxide (about 60% in the inhaled air). This gas mixture provides painless death. After incision of the abdominal wall and removing the subcutaneous fatty tissue and the abdominal wall muscle layers, fragments of abdominal skin of the animal have been collected. The remaining part of skin was subdivided into 2 cm^2^ pieces, which were submerged and incubated in the appropriate solution, according to the experimental protocol (see Table 1).

### 2.2. Chemicals

The following chemicals were used for the experiment:

- RH: Ringer solution: basic solution with iso-osmotic properties (K^+^ 4.0 mM; Na^+^ 147.2 mM; Ca^2+^ 2.2 mM; Mg^2+^ 2.6 mM; Cl^−^ 160.8 mM; Hepes 10.0 mM), which was adjusted to pH 7.4 under the control of a pH-meter
- Amiloride hydrochloride hydrate: an inhibitor of transepithelial sodium ions transport; amidynoamid acid, 3,5-diamino-6-chloro-2-carboxylic acid; 266.09 g/mol (Sigma-Aldrich),
- AMI: amiloride (0.1 mM) dissolved and diluted in Ringer solution,
- Bumetanide: an inhibitor of transepithelial chloride ions transport; 3-aminosulfonyl-5-butylamino-4-phenoxybenzoic acid (Sigma-Aldrich),
- B: bumetanide (0.1 mM) dissolved in DMSO (dimethylsulfoxide) and diluted in Ringer solution,
- Amiloride in bumetanide (0.1 mM) dissolved in Ringer solution. It is an inhibitor of transepithelial sodium and chloride ions transport.

Mineral compounds (KCl, NaCl, CaCl_2_, and MgCl_2_) were purchased from POCH, Poland.

### 2.3. Preparation of Ethanol Extract of Propolis

The material for research was ethanol extract of propolis (EEP). Propolis was produced by honeybees from the apiary in Białośliwie (Wielkopolska, Great Poland). Hand-collected propolis came from the collection of 2016 year and was desiccated and kept in dark before processing. The portion of 50 g of propolis was put into black bottle. Next 350 cm^3^ of 95% ethanol-Grain Luxury Spirit (CEDC International, made by its branch Polmos Białystok, Poland) and 150 cm^3^ water were added. Propolis was submitted to 21 days of extraction in order to obtain its ethanol extract. The black bottle was placed in laboratory at room temperature and its content was mixed two times a day for 21 days. After that time brown, rough particles were removed from propolis extract by filtering (sterile bandage MATOCOMP, Dressing Material Factories, Toruń, Poland).

### 2.4. Preparation of Propolis Ointment

The vessel has been instilling a portion of 100 ml ethanol extract of propolis and placed into hot water bath until dense liquid was obtained. Next, the dark, brown, dense liquid was added to 100 g colourless cosmetic white petrolatum-*Vaselinum album* (Pharmaceutical Laboratory by AVENA, Osielsko, Poland). Having mixed white petrolatum with evaporated propolis extract, the yellow ointment was formed.

### 2.5. Experimental Procedure

The skin fragments were taken from laboratory animals. After cutting, the animal skin fragments were incubated for 30 minutes (1 ml ethanol extract of propolis, Ringer solution, propolis ointment) and placed in the adapter. This apparatus has two rubber gaskets lubricated with silicone grease, to prevent tissue damage. The adapter was placed in an Ussing chamber composed of two half cells between which the examined tissue has been put. After replenishing chambers with proper liquid, water hoses were shut down with metal snaps. Two pairs of chloride-silver electrodes, connected to measuring set (linked with computer), were used to check voltage and current, to measure the resistance. Electrophysiological parameters were tested using epidermal tissue under mechanical and mechanical-chemical stimulation (Table 1.) These parameters were checked with the presence of chloride ions transport blockers (bumetanide) and blockers of Na^+^ ions and Cl^−^ ions by using both substances (amiloride and bumetanide). Isolated tissue was rinsed with reagents by the use of peristaltic pump to maintain a constant flow rate of 3 ml per 15 seconds. The experimental procedure, conducted at the temperature 23±2°C, lasted about 1 hour. This time was provided for a single tissue fragment.

### 2.6. Data Analysis

The experimental data were recorded using the experimental protocol. EVC 4000 (WPI, USA) apparatus was used to measure voltage and resistance. It was connected to a computer data acquisition experimental system MP 150 (Biopac, USA) and translated using AcqKnowledge 3.8.1. (WPI, USA) software. Electrophysiological parameters were compared in Microsoft Excel 2016 (Microsoft, Poland). The statistical data relationship was performed using STATISTICA 13.1 (Statsoft, Poland). Shapiro-Wilk's test was applied for the compatibility estimation of the assessed parameters distribution with normal distribution. All results were expressed as mean ± SD/median/interquartile range. All measurements were interpreted in the three groups using a nonparametric Wilcoxon signed-rang test and Mann Whitney U test. Two-tailed P value of < 0.05 was considered statistically significant.

## 3. Results

### 3.1. The Comparison of Electrophysiological Parameters of 1ml EEP in 100 ml Water (Group I), Ringer's Solution (Group II), and Ringer's Solution with Propolis Ointment (Group III).

**Figure 1:**
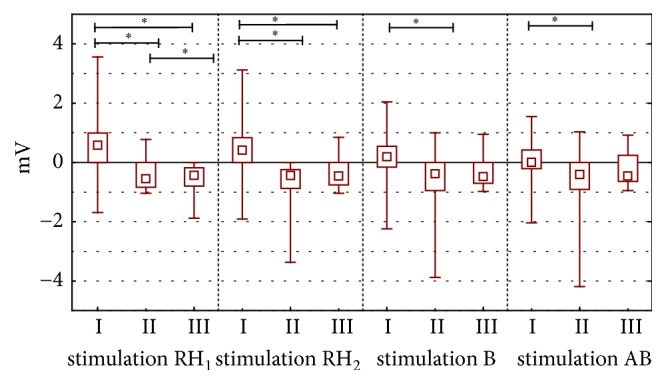
The electrical potential in stationary conditions before stimulation. The figure shows arrangement the electrical potential value before individual stimulation. The experiment were performed according to scheme stimulations by Ringer solution (RH), bumetanide (B), amiloride (AMI) and amiloride with bumetanide (AB). The time of each stimulation was 15 seconds. The figure shows also first and second simulation by Ringer solution (stimulation RH_1_ and stimulation RH_2_) and stimulation by bumetanide (stimulation B) stimulation by amiloride in bumetanide (stimulation AB).* Abbreviations. ∗*p < 0.001, RH: Ringer fluid, stimulations 1 and 2, B: bumetanide solution (0.1 mM), AB: a mixture of amiloride and bumetanide solutions (0.1 mM). I: long-term incubation in 1 ml ethanol extract of propolis dissolved in 100 ml water, II: undisturbed ion transport, and III: long-term incubation in propolis ointment;

The values of electric potential of experimental group I and II before mechanical stimulation RH_1_ (p<0.001), RH_2_ (p<0.001), bumetanide (p=0.004), and amiloride with bumetanide (p=0.018) were different with statistical significance. Between experimental groups II and III this difference is either visible for stimulation with RH_1_ solution (p<0.001) (Figure 1).

**Figure 2:**
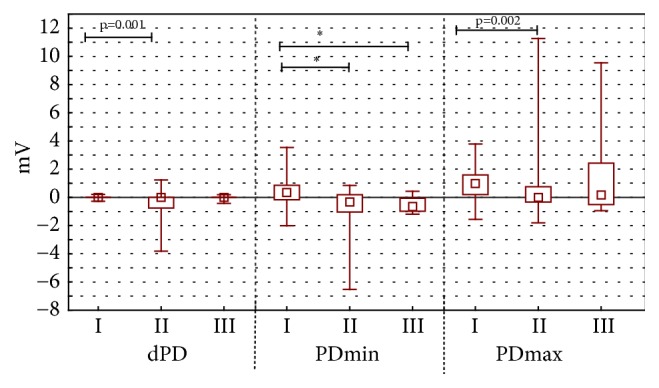
The mean, minimal and maximal transepithelial potential difference value for mechanical stimulation RH_1_. The figure shows arrangement the difference of electrical potential, its minimal and maximal value during first stimulation by Ringer solution (stimulation RH_1_). The time of stimulation was 15 seconds.* Abbreviations. ∗*p < 0.001. I: long-term incubation in 1 ml ethanol extract of propolis dissolved in 100 ml water, II: undisturbed ion transport, III: long-term incubation in propolis ointment, dPD: difference of electrical potential values during RH_1_ stimulation (mV), PD_min_: minimal transepithelial electrical potential during RH_1_ stimulation (mV), and PD_max_: maximal transepithelial electrical potential during RH_1_ stimulation (mV).

**Figure 3:**
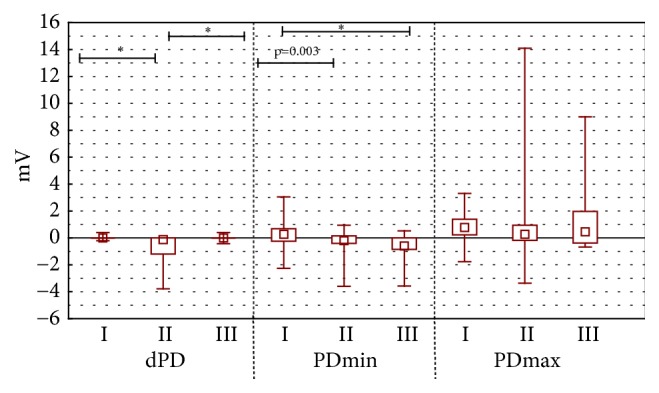
The mean, minimal, maximal transepithelial potential difference value for mechanical stimulation RH_2_. The figure shows arrangement the difference of electrical potential, its minimal and maximal value during second stimulation by Ringer solution (stimulation RH_2_). The time of stimulation was 15 seconds.* Abbreviations. ∗*p < 0.001. I: long-term incubation in 1 ml ethanol extract of propolis dissolved in 100 ml water, II: undisturbed ion transport, III: long-term incubation in propolis ointment, dPD - difference of electrical potential values during RH_2_ stimulation (mV), PD_min_: minimal transepithelial electrical potential during RH_2_ stimulation (mV), and PD_max_: maximal transepithelial electrical potential during RH_2_ stimulation (mV).

The difference of stationary potential during the stimulation by RH_1_ solution (Figure 2) and RH_2_ solution (Figure 3) for experimental groups I and II was as observable (p<0.001) as during RH_2_ (Figure 3) stimulation in experimental groups II and III (p<0.001).

**Figure 4:**
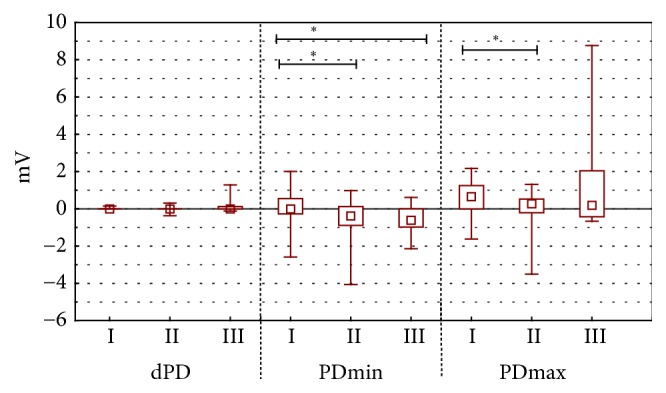
The mean, minimal, maximal transepithelial potential difference value for chemical stimulation B. The figure shows arrangement the difference of electrical potential, its minimal and maximal value during stimulation by bumetanide (stimulation B). The time of stimulation was 15 seconds. Statistical significance between groups was marked as *∗*p < 0.001.* Abbreviations. ∗*p < 0.001. I: long-term incubation in 1 ml ethanol extract of propolis dissolved in 100 ml water, II: undisturbed ion transport, III: long-term incubation in propolis ointment, dPD: difference of electrical potential values during bumetanide stimulation (mV), PD_min_: minimal transepithelial electrical potential during bumetanide stimulation (mV), and PD_max_: maximal transepithelial electrical potential during bumetanide stimulation (mV).

Maximal value of transepidermal electric potential was changing in groups I and II during the stimulation by RH_1_ solution (p=0.002) (Figure 2) and bumetanide (p<0.001) (Figure 4).

**Figure 5:**
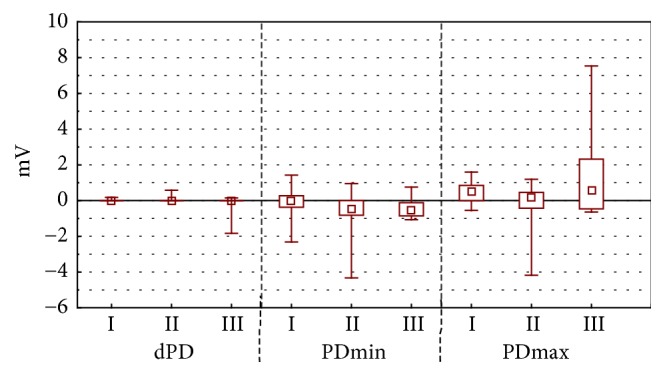
The mean, minimal, maximal transepithelial potential difference value for chemical stimulation AB. The figure shows arrangement the difference of electrical potential, its minimal and maximal value during stimulation by amiloride with bumetanide (stimulation AB). The time of stimulation was 15 seconds. In this experiment no statistically significant changes.* Abbreviations. ∗*p < 0.001. I: long-term incubation in 1 ml ethanol extract of propolis dissolved in 100 ml water, II: undisturbed ion transport, III: long-term incubation in propolis ointment, dPD: difference of electrical potential values during amilloride in bumetanide solution stimulation (mV), PD_min_: minimal transepithelial electrical potential during amiloride in bumetanide solution stimulation (mV), and PD_max_: maximal transepithelial electrical potential during amiloride in bumetanide solution stimulation (mV).

During the experiment no statistically significant differences of minimal and maximal value of electric potential occurred and neither did the differences in value of electric potential during amiloride with bumetanide (Figure 5) stimulation between experimental groups I and II and II and III.

### 3.2. The Comparison of Electrophysiological Parameters between Group I (1ml EEP in 100ml Water) and Group III (Propolis Ointment)

**Figure 6:**
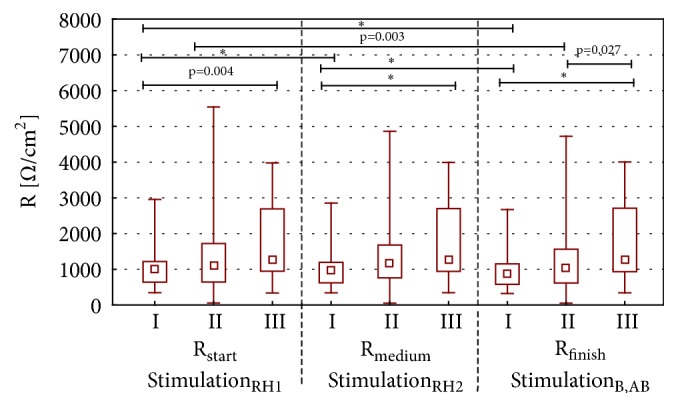
The resistance for I, II, and III group during RH_1_, RH_2_, B, and AB stimulation. This figure shows arrangement the electrical resistance for first and second simulation by Ringer solution (stimulation RH_1_, stimulation RH_2_), stimulation by bumetanide (stimulation B), and stimulation by amiloride with bumetanide (stimulation AB).* Abbreviations. ∗*p < 0.001. I: long-term incubation in 1 ml ethanol extract of propolis dissolved in 100 ml water, II: undisturbed ion transport, III: long-term incubation in propolis ointment, R_start_ (RH_1_): transepithelial resistance for first Ringer solution stimulation, R_medium_ (RH_2_): transepithelial resistance for second Ringer solution stimulation, and R_finish_ (B, AB): transepithelial resistance for bumetanide solution and mixture of amiloride with bumetanide solution stimulations.

During the stimulation with RH_1_ and RH_2_ the solution, propolis ointment and 1ml of EEP in 100 ml of water were showing different stationary potentials (PDbefore) (Figure 1) and minimal values of transepidermal electric potential (PDmin). These differences were statistically significant (value p<0.001 for all measured parameters (Figures 2 and 3). Statistically significant difference of electric resistance (R) values between groups I and III for RH_1_, RH_2_, bumetanide, and amiloride with bumetanide solution stimulations (Figure 6) was noticed (respectively, p=0.004, p<0.001, and p<0.001). Minimal potential during bumetanide solution stimulation showed statistically significant difference for both propolis solutions (respectively, p<0.001 and p<0.001) (Figure 4). There were no differences of electrical potential (dPD) values during stimulation as well as maximal and minimal potential values during amiloride with bumetanide solution (Figure 5) stimulation between experimental groups I and III.

### 3.3. Value of Electrical Resistance between Experimental Groups

Difference between initial value of electrical resistance (after RH1 stimulation) and final value of electrical resistance (after B, AB stimulation) for Ringer solution (p=0.003) and 1ml of EEP in 100ml of water (p<0.001) was statistically significant (Figure 6). Changes of electrical value were essentially different for propolis tincture (1ml of EEP in 100ml of water) during each stage of experiment (p<0.001). During the experiment described above no statistically significant differences of electrical resistance for propolis ointment were found as well as between Ringer solution and 1ml of EEP in 100 ml of water. However, the differences of electrical resistance were important between propolis ointment and propolis tincture (p<0.001 for medium and finish value of resistance and p=0.004 start value of resistance). The value of final resistance differs significantly for Ringer solution and propolis ointment (p = 0.027, Table 2).

## 4. Discussion

Ion transport in epidermal cells is taking place through ion channels, ion pumps and transporters localized on apical and basolateral surface of cell membrane. In research, the modification of ion channels permeability is achieved by mechanical and chemomechanical surface stimulation. Transport protein system permits the changes in ions layout in both sides of tested epidermal tissue and thereby generates and keeps transepidermal electrical potentials difference on epidermal surface. Difference of electrical potential shows functioning of electric field on the surface of epidermis and its protective properties [30, 31].

Our research shows the influence of long-term incubation with substances containing propolis and short-term stimulation using the Ringer solution, bumetanide, amiloride with bumetanide. In order to check the effect of substances contained in propolis on sodium and chloride ion transport located in the skin, we used bumetanide and amiloride with bumetanide. Bumetanide stimulation was made to prevent transepidermal chloride ion transport through blocking basolateral cotransport mechanism. During the stimulation with bumetanide, the differences of electrical parameters value between group with propolis tincture and Ringer solution group were observed. Skin incubation with 1ml of EEP contained in 100ml of H_2_O and blockage of chloride channels after use of bumetanide were influencing better hydration of skin cells and having positive effect on skin layers hydration. The value of the electrical resistance of the skin is affected by skin hydration, its thickness, damage to continuity, the presence of inflammation, and the degree of blood supply to the dermis [32]. As a result of stratum corneum damage, a decrease of electrical skin resistance was observed. The resistance was reduced by direct contact of electric current with the lower layers of the epidermis and a rapid increase in the flow of electric current occurred [32, 33]. During the experiment with the use of 1 ml ethanol extract of propolis dissolved in 100 ml water, a decrease in electrical resistance was observed, which occurred as the result of damage to the skin surface. Damage to the skin surface causes a greater impact of irritants and an increased transepidermal water escape from its surface. In dehydrated skin, a decrease in electrical capacity and increase of electrical resistance was observed. The skin is subject to reversed proportions, and the skin's electrical capacity decreases, while its electrical resistance increases [33]. There was no subsequent increase in electrical resistance in the experiment, while a further decreasing electrical resistance was observed. Good skin hydration is characterized by increasing skin electrical capacity and decreasing electrical resistance. In our research bumetanide, which is an inhibitor of transepithelial chloride ions transport, was used. In the same moment when chloride ions transport was inhibited, an increased influx of sodium ions to skin cells and inflow of water into skin cells occurred in the experiment. As a result of the inflow of water, an increased level of skin hydration was observed. The decreasing electrical resistance in the course of the test indicates properly skin hydration. In the scientific literature there are no publications about effects of cosmetic preparations on ions transport in human skin or rabbit skin. On the other hand there are a lot of publications about ions transport in isolated rabbit trachea, respiratory epithelium, large intestine or frog skin. Effect of irritant substance such us capsaicin and its influence on ion transport was described in the publication of Hołyńska-Iwan et al. (2018) [34]. Similarly to the results of the obtained experiment, capsaicin causes some modification of the sodium ions transport, which in turn causes their inflow into the skin cells can improve skin hydration. In addition, capsaicin may modify the action of epithelial sodium channels. Due to the different nature of the tested substances and another experimental procedure, their results are incomparable.

The use of propolis tincture on skin surface influenced sodium ions transport. During amiloride solution and bumetanide solution stimulations, the changes in values of electrophysiological skin parameters did not occur. Propolis ointment did not influence both sodium and chloride ions transport in tested rabbit's skin fragments. The changes of electrophysiological parameters of rabbit's skin during experiments proved that tested epidermal tissue, derived from laboratory animals, was reactive. The skin, thanks to multilevel, bipolar structure, tight intracellular connections, abundant amount of lipids, and keratin, was showing high value of electrical resistance [33]. The values of electrical resistance observed during the study were higher than values obtained for other tissues incubated with the use of Ussing chamber. Data presented in the article written by Wolska et al. [35] illustrate lower values of electrical resistance of experimental rabbits tissues after Ringer solution stimulation in comparison to the results obtained in our study. The value of electrical resistance in Wolska et al. study for rabbits trachea was 129.0 Ohm/cm^2^ and for intestine 273 Ohm/cm^2^ [35].

The analysis of influence of propolis preparations on value of electrical resistance during our study was proving discrepancy of results between control group (Ringer solution) and group with propolis ointment and 1ml of ethanol extract of propolis. Propolis ointment and ethanol extract of propolis dissolved in water was showing statistically important difference of electrical resistance. In our study, the use of 1 ml ethanol extract of propolis dissolved in 100 ml water causes a decrease in electrical resistance during the experiment. The use of an propolis ointment did not cause changes in electrical resistance, as well as the use of Ringer solution.

Differences between these values result from different effect of both preparations on skin surface and have influence on their usage. Petrolatum, through occlusive properties, prevents transepidermal water loss and reduces vaporization from skin surface that help to keep water inside its cells. This substance is hydrophobic, insoluble in water. It does not create unpassable barrier and penetrate through cortex layer of epidermis and makes the regeneration after the damage possible despite of occlusive properties [36, 37]. The occlusive properties of petrolatum prevented damage to the skin surface. Long-term application of petrolatum is not recommended because of forming a film on the skin, slowing down gas exchanges and metabolism of skin, and also decreasing water evaporation and increasing tendency to keep filth and pollution on the skin surface [38]. The result of its occlusive influence is an increase of cortex layer hydration level and reduction of transepidermal water loss by 43%.

The occlusive effect induces comedogenic properties. There are the differences between humans and animal examples. The rabbit skin is much more prone to comedogenicity in comparison to the human skin. Vaseline is safe to use in cosmetics thanks to their limited penetration. It improves the skins softness, but works only on superficial epidermis layers. Petroleum jelly prevents penetration of hydrophilic substances through skin [39]. In our study, petrolatum with ethanol extract of propolis was used to obtain propolis ointment. Ethanol extract is a hydrophilic substance. Petrolatum, through its properties of unable penetration of hydrophilic substances, affects the surface of epidermis. Propolis does not penetrate reaching the depths of skin, so it affects only the skin surface. Ethanol, a substrate of second propolis preparation used in our study, strengthens influence of ethanol on transepidermal ion transport and enables the penetration of propolis in skin depths. Ethanol causes an increase of concentration gradient through enhancement of active compound solubility, as a main driving strength transepidermal transport processes. Important element of chemical composition of cosmetics and their later influence on skin surface are the so-called promotors of transepidermal transport. Ethanol is one of the most commonly used sorption promotors. Sorption promotors, through their influence on lipid matrix of epidermis, speed up and facilitating the diffusion through stratum corneum layer of epidermis. Ethanol extract of propolis dissolved in water penetrates deeper in comparison to propolis ointment [40]. Additional factor, which influences the electrical resistance difference between experimental groups I and II, is a manner of preparation of propolis ointment, which is created through propolis extract evaporation in water bath.

The difference in the effect of these substances on electrical resistance is due to the use and cosmetic properties of petrolatum. It is the only cosmetic ingredient which is different in research groups—1 ml ethanol extract of propolis dissolved in 100 ml water and propolis ointment.

Important differences in value of electrical resistance occur in our study during each 15-second stimulation by RH, AMI, B, and AB solutions. Initial raise of electrical resistance for Ringer solution and propolis ointment in above described study can be caused by reduced water loss. Propolis tincture causes reduction of electrical resistance value through deeper skin penetration and progressive changes in hydration and adhesiveness of skin cells.

It seems that the rabbit skin, which was used in experimental model, is appropriate skin equivalent to study the effects of various substances on epidermal tissue properties in comparison to the human skin [41, 42]. Experimental model in reference to the human skin is not perfect. Differences are the result of interspecific variety. The study of Jirova et al. [42] confirms that rabbit skin is more sensitive than human skin. From 16 chemical substances classified as irritant for rabbits' skin, five were significantly irritant for the same human tissue. Reinferath et al. [43] suggest that with hair density growth in animals, permeability of tissues also increases. Our study suggests that human skin has lower transepidermal permeability for xenobiotics in comparison to skin fragments excised from abdominal surface of experimental animals [43]. Influence of preparation containing propolis on electrophysiological parameters rabbit skin is a referential model to specify the influence on electrophysiological parameters of human skin considering existing interspecific differences. Studies show that the manner of substance preparation and its penetration depth influence the ion transport in the skin and the value of electrical resistant. This research is a preliminary, pioneering study demonstrating the effect of propolis on ion transport. There is a need for further research in which propolis component affects the transport of ions.

## 5. Conclusions

1. Model skin study in Ussing chamber allows valueing fast, several-second changes of transepidermal ion transport, which are important for transport of various substances into skin and their positive effects.
2. The studies of transepidermal electrical potential difference in connection to the use of sodium and chloride ions transport inhibitors allow checking the influence of propolis containing substances on the chosen transport of ions.
3. Ethanol propolis extract dissolved in water influences transepidermal sodium ions transport.
4. Propolis ointment does not influence transepidermal sodium ions transport.
5. The way of preparation cosmetics, which contain propolis, has effects on transepidermal ions transport in the rabbit's skin.
6. The value of skin electrical resistance is changing with penetration depth of active propolis substances contained in cosmetics.

## Figures and Tables

**Table 1:** The experimental table taking into account the experimental groups, incubation solutions, type of stimulation, and electrophysiological parameters.

**Group**	**Pre- incubation fluid (30 min)**	**Incubation fluid (30 min)**	**Mechanical stimulation fluid**	**Chemical stimulation fluid (15s)**	**Electrophysiological parameters **
**I group:** Long-term incubation in 1 ml ethanol extract of propolis in 100 ml water *N =22*	1 ml EEP in 100 ml water + RH	RH	RH	B AB	PDmin PDmax R dPD PD
**II group:** Undisturbed ion transport *N =22*	RH	RH	RH		
**III group:** Long- term incubation in propolis ointment *N =18*	propolis ointment + RH	RH	RH		

*Abbreviations.* N: number of specimens, RH: Ringer solution, B: bumetanide (0.1mM) in Ringer solution, AB: amiloride in bumetanide (0.1mM) in Ringer solution, PD: transepithelial electrical potential differences in stationary conditions before and after stimulation (mV), dPD: changes of electrical potential differences during stimulation (mV), PDmin: minimal transepithelial electrical potential (mV), PDmax: maximal transepithelial electrical potential (mV), and R: transepithelial resistance (Ohm/cm^2^).

**Table 2:** The comparison of transepithelial electrical resistance between RH solution, 1 ml ethanol extract of propolis in 100 ml of water, and propolis ointment.

Stimulation	(R Ω/cm^2^) median value			U Mann Whitney test (p value)		
	RH	Propolis ointment	1 ml EEP in 100 ml water	RH comparison with propolis ointment	RH comparison with 1 ml EEP in 100 ml water	Propolis ointment comparison with 1 ml EEP in 100 ml water
R_start_ (stimulation RH_1_)	1119	1269	1018	0.064	0.211	0.004

R_medium_ (stimulation RH_2_)	1184	1273	966	0.055	0.082	< 0.001

R_finish_ (stimulation B, AB)	1049	1257	883	0.027	0.175	< 0.001

Wilcoxon test (p value)						

	RH	Propolis ointment	1 ml EEP in 100 ml water			

R_start_ -R_medium_	0.345	0.806	< 0.001			

R_start_ - R_finish_	0.003	0.507	< 0.001			

R_medium_-R_finish_	0.085	0.148	< 0.001			

***Abbreviations.***R_start_ (stimulation RH_1_): the value of electrical resistance after the first stimulation by Ringer solution; R_medium_ (stimulation RH_2_): the value of electrical resistance after the second stimulation by Ringer solution; R_finish_ (stimulation B, AB): the value of electrical resistance after stimulation by bumetanide and amiloride in bumetanide; R_start_ - R_medium_: the comparison of the value of the electrical resistance after first stimulation by Ringer solution with the value of electrical resistance second stimulation by Ringer solution; R_start_ - R_finish_: the comparison of the value of the electrical resistance after first stimulation by Ringer solution with the value of electrical resistance second stimulation by bumetanide and amiloride in bumetanide; R_medium_-R_finish_: the comparison of the value of the electrical resistance after first stimulation by Ringer solution with the value of electrical resistance second stimulation by bumetanide and amiloride in bumetanide.

## Data Availability

The data used to support the findings of this study are included within the article.
